# Comparison of Different Feature Sets for TLS Point Cloud Classification

**DOI:** 10.3390/s18124206

**Published:** 2018-11-30

**Authors:** Quan Li, Xiaojun Cheng

**Affiliations:** College of Surveying and Geo-Informatics, Tongji University, Shanghai 200092, China; cxj@tongji.edu.cn

**Keywords:** terrestrial laser scanning, point cloud, classification, intensity, feature set, supervoxel, random forest

## Abstract

Point cloud classification is an essential requirement for effectively utilizing point cloud data acquired by Terrestrial laser scanning (TLS). Neighborhood selection, feature selection and extraction, and classification of points based on the respective features constitute the commonly used workflow of point cloud classification. Feature selection and extraction has been the focus of many studies, and the choice of different features has had a great impact on classification results. In previous studies, geometric features were widely used for TLS point cloud classification, and only a few studies investigated the potential of both intensity and color on classification using TLS point cloud. In this paper, the geometric features, color features, and intensity features were extracted based on a supervoxel neighborhood. In addition, the original intensity was also corrected for range effect, which is why the corrected intensity features were also extracted. The different combinations of these features were tested on four real-world data sets. Experimental results demonstrate that both color and intensity features can complement the geometric features to help improve the classification results. Furthermore, the combination of geometric features, color features, and corrected intensity features together achieves the highest accuracy in our test.

## 1. Introduction

Terrestrial laser scanning (TLS) devices have been widely used to quickly acquire 3D spatial information of large-scale urban scenes. The classification of point clouds is a key step for utilizing the information effectively. The common workflow of 3D point cloud classification involves neighborhood selection, feature selection and extraction, and classification of points based on the respective features [1]. The neighborhood selection has always been the focus of many studies. Many previous studies focused on point-wise classification techniques, in which features for every point are calculated by using the points within its neighborhood. The spherical neighborhood and cylindrical neighborhood are often used. However, these neighborhoods with fix-bound radiuses may be inappropriate for TLS point clouds because of their varying point densities. The K-nearest neighbors can provide irregular neighborhood sizes depending on the density of its point cloud and its flexibility. However, it requires a high computation complexity. To improve the computational efficiency, some authors have proposed voxel- or supervoxel-based neighborhoods for feature extraction. In the study of Lim and Suter, they first segment the point cloud into supervoxels as support regions, after which a multiscale conditional random field is used to classify a TLS point cloud [2]. Ramiya et al. uses supervoxel-based segmentation to segment the point cloud data first, and then uses different machine-learning algorithms to label the point cloud [3]. Wang et al. proposes an object detection method based on supervoxel neighborhoods with a Hough forest frame; their method have shown good results with high efficiency [4]. Plaza-Leiva et al. utilizes voxel-based neighborhoods to extract features. In their method, the points in each voxel are assigned the same class. Their experimental results have proven the feasibility of voxel-based neighborhoods in 3D point cloud classification and its high computational efficiency [5].

Feature selection and extraction has also been the research interest for many previous studies. The features commonly used are often related to the properties of geometry [6]. Besides the geometric information, most TLS systems also record the intensity information, which is considered to be an important measurement of the spectral property of a scanned surface. It can also serve as an additional feature in point cloud classification. There exist several studies on airborne laser scanning (ALS) and mobile laser scanning (MLS), which have used intensity data for classification. Song et al. first used intensity data for ALS point cloud classification; they conclude that intensity can be used for ALS land-cover classification [7]. In the study of Zhou et al., the height and intensity data were integrated for land-cover classification; the experiment results proved the effectiveness of height and intensity data [8]. Zhang et al. used thirteen features that contain geometry, intensity, topology, and echo characteristics to train the support vector machine (SVM) classifier for ALS point cloud classification [9]. In most MLS studies, intensity has been used for the detection of road markings and road signs [10,11,12]. However, for TLS point clouds, there are very limited studies that have used intensity data for point cloud classification. Lim et al. combined color and intensity with geometric features to form a feature set used for their supervised learning model [2], whereas Wang et al. combined the median of intensity with other 27 features in a Hough Forest frame for object detection in a TLS point cloud [4]. In their recent study, Hackel et al. refrained from using intensity information, claiming that it does not improve the classification in their tests [13]. This may be due to the fact that the original intensity data was affected by several factors which may have degraded the classification performance, not to mention that intensity correction is always recommended before fully utilizing the intensity information [14]. In addition, many TLS systems are also equipped with digital cameras which can acquire corresponding color information for each point. Several previous studies have also used color information for TLS point cloud classification. Li et al. used geometric features, the mean RGB color, and the LAB values of that mean RGB in order to train a linear SVM classifier for TLS point cloud classification [15]. Aijazi et al. incorporated RGB and intensity values on classification and found that intensity with RGB values performs better in classification than RGB color alone [16].

In this paper, we carefully investigate the influence of several different feature sets on TLS point classification. We first use supervoxel-based neighborhood for feature extraction considering its computational efficiency. To the best of our knowledge, this is the first time a supervoxel is used as the support region in a point-based classification. The features of each point within one supervoxel are calculated using all these points inside the supervoxel; hence, every point in one supervoxel has the same features, and all points are assigned the same class label. In order to improve both accuracy and efficiency, a novel supervoxel segmentation method proposed by Lin et al. is used instead of using commonly-used methods [17]. The features we investigated include geometric features, color features, and intensity features. Moreover, the original intensity is corrected to eliminate range effect, therefore the corrected intensity features are also extracted and used. To our knowledge, there have never been any studies which have analyzed corrected intensity features on TLS point cloud classification. These features are then combined together to compose different feature sets which are further applied to the Random Forest classifier for classification. The comparative performance analysis is carried out on four real-world data sets. Experiment results have demonstrated that both color and intensity can complement geometric information to help improve the classification results, and geometric features combined with color and corrected features achieve the best classification accuracy in our test.

The remainder of this paper is organized as follows: the methodology is described in Section 2, the experiments conducted are described in Section 3 followed by a discussion, and finally, conclusions follow in Section 4.

## 2. Methodology

**Figure 1:**
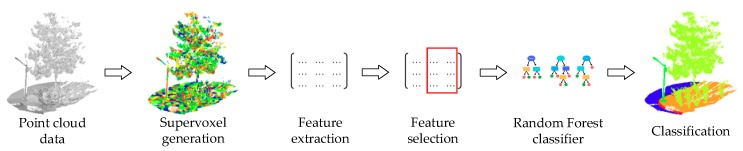
The general framework of this study.

The general framework of this study is given in Figure 1. The original point cloud data acquired are first over-segmented into spatially consistent supervoxels. Then, different features are extracted based on the supervoxel neighborhood. These features mainly consist of geometric features, color features, and intensity features. Note that before intensity feature extraction, the original intensity of the point cloud is corrected for range effect in order to derive the corrected intensity data. Therefore, the intensity features comprise both original and corrected intensity features. After the feature extraction, different features are combined together to compose different feature sets, which are then used for training the random forest classifiers in the training stage and for classification in the prediction stage. Finally, the recall, precision, F1-score, and overall accuracy are used to evaluate the classification performance.

### 2.1. Supervoxels Generation

A supervoxel groups 3D points into perceptually meaningful clusters with high efficiency. According to the definition of supervoxel, points within a supervoxel must have similar features and be spatially connective. Supervoxels have long been preferred as basic processing units instead of original points in point cloud applications. Voxel cloud connectivity segmentation (VCCS) is one of the commonly-used supervoxel generation methods [18]. However, when the density of a point cloud varies a lot, VCCS cannot preserve the boundaries well, which leads to under-segmentation errors and classification errors. In this paper, we used the method proposed by Lin et al., which can provide better-preserved object boundaries and lower under-segmentation errors [17]. In their method, they formalize supervoxel generation as a subset selection problem which involves an explicit energy function. They also develop a simple but effective method to minimize the energy function for a subset selection which does not need to select seed point. Moreover, this method is more computationally efficient than most of the state-of-the-art supervoxel methods. In this study, the supervoxel was selected as a neighborhood for feature extraction. The features of a point in one supervoxel were calculated using all of these points in this supervoxel, meaning the features of all points within this supervoxel were the same, and all points were assigned the same class label within one supervoxel.

### 2.2. Feature Sets Extraction

After supervoxel generation, different features are extracted based on the supervoxel neighborhood. Feature selection and extraction constitute the essential part of point cloud classification, and their performance plays a decisive role in classification results [19]. In this paper, we carefully select three types of features for classification: geometric features, color features, and intensity features.

Before the feature extraction, intensity correction is first conducted. Intensity correction means converting the original intensity data into a corrected value which is proportional or equal to target reflectance [20]. The original intensity is affected by factors including the scanning geometry, the scanner mechanism, and the surrounding environment. The scanning geometry is a major factor which includes range and incidence angles [21]. The correction of these two effects has been the focus of many previous studies [22,23,24]. Many studies also found that the intensity data of TLS does not follow the LiDAR equation in the near range and different TLS systems may result in different intensity–range relations [21,23,24,25]. Therefore, the data-driven model has been proven to be more appropriate for TLS intensity data correction. The effect of incidence angles is more complicated than the range effect, because the characteristics of the scanned surface need to be considered. Most previous studies simply assumed the scanned surface to be a perfect Lambertian surface which is not accurate, because both diffuse and specular reflections exist in natural surfaces. The correction results may be unsatisfactory when an inappropriate surface reflection model is used. Therefore, in this paper, only the range effect has been considered and corrected. According to the intensity correction definition, the range corrected intensity data $I_{C}$ (note: $I_{C}$ still relies on other factors, such as incidence angle and scanner mechanism effects) can be derived as follow:

(1) $$I_{C}=I\times \frac{f\left(R_{s}\right)}{f\left(R\right)}$$

where I is the original intensity, R is the distance, $R_{s}$ is the selected reference distance, and f(·) is the approximated intensity as a function of range, its specific form being derivable through certain experiments. In Equation (1), the range effect is removed by normalizing the intensity data into a user-defined standard range. 

Formally, a point cloud can be written as $P=\left\{x_{i},{\;y}_{i},{\;z}_{i},{\;I}_{i},{\;R}_{i},{\;G}_{i},B_{i}\right\}$, consisting of the coordinates $x_{i},{\;y}_{i},{\;z}_{i}$ of the 3D point, the intensity data $I_{i}$ and the RGB values $R_{i},{\;G}_{i},B_{i}$. The derived supervoxel neighborhood serves as the basis for feature extraction. In this study, we extracted three types of features, namely geometric features, color features, and intensity features. The geometric features are mainly comprised of covariance features, which are derived from the normalized eigenvalues $\lambda_{1,\;2,\;3}$ of the 3D structure tensor [26,27]. These eigenvalues are sorted as $\lambda_{1}\geq \lambda_{2}\geq \lambda_{3}$. The covariance features are quite useful in the representation of the local geometric shape in a certain neighborhood. Besides covariance features, three other features derived from the supervoxel neighborhood are given by mean *z* value, *z* variance, and maximum *z* difference. The considered color feature set comprised 12 features: mean R, G, and B; R, G, and B ratio; R, G, and B variance, and maximum R, G, and B difference. The considered intensity feature set comprises three features which involve mean intensity, intensity variance, and maximum intensity difference. All these features are listed in Table 1. Note that the color features and intensity features are normalized to a range between 0 and 1 before being applied to the Random Forest classifier.

### 2.3. Classifier

In this paper, the Random Forest classifier was used for the classification of the TLS point cloud [28]. A random forest provides a good trade-off with respect to both accuracy and computational efficiency. In addition, it has been proved to be successfully applied to point cloud classifications [29,30]. A random forest is an ensemble algorithm that creates a set of decision trees from a training set’s randomly selected subsets. It then aggregates the votes from different decision trees to decide the final class of the test object. It therefore has a high predictive accuracy and control over-fitting. In order to analyze the performance of different feature sets on the classification results, we trained six random forest classifiers. They were trained with 11 geometric features, 11 geometric features combined with 3 original intensity features, 11 geometric features combined with 3 corrected intensity features, 11 geometric features combined with 12 color features, 11 geometric features combined with 3 original intensity features and 12 color features, and 11 geometric features with 12 color features and 3 corrected intensity features, respectively.

### 2.4. Performance Evaluation

In this paper, we used four commonly used measures for our evaluation: recall, precision, overall accuracy, and an F1-score. Recall represents a measure of completeness or quantity, precision represents a measure of exactness or quality, overall accuracy indicates the overall performance of the classification result, and the F1-score is the harmonic mean of recall and precision. All four evaluation measures are described below in Equations (2)–(5).

(2) $$\mathrm{Recall}=\frac{\mathrm{TP}}{\mathrm{TP}+\mathrm{FN}}$$

(3) $$\mathrm{Precision}=\frac{\mathrm{TP}}{\mathrm{TP}+\mathrm{FP}}$$

(4) $$\mathrm{Overall}\;\mathrm{accuracy}=\frac{\mathrm{TP}+\mathrm{FN}}{\mathrm{TP}+\mathrm{FP}+\mathrm{TN}+\mathrm{FN}}$$

(5) $$F1\;\mathrm{score}=2\times \frac{\mathrm{Recall}\times \mathrm{Precision}}{\mathrm{Recall}+\mathrm{Precision}}$$

where TP, FN, FP, and TN denote the number of true positives, false negatives, false positives, and true negatives, respectively. 

## 3. Experiment Results and Discussion

### 3.1. Data Sets

**Figure 2:**
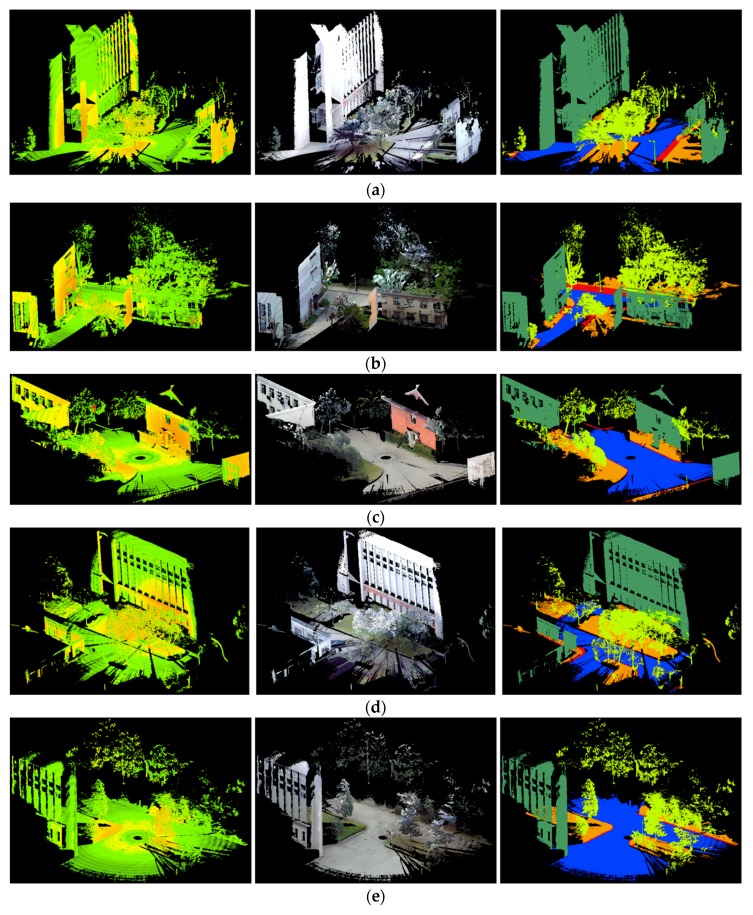
Intensity values (left), RGB colors (middle), and ground truth (right) for: (**a**) training set, (**b**) testing set 1, (**c**) testing case 2, (**d**) testing case 3, and (**e**) testing case 4. Legend for labels: ground façade pole tree vegetation curb.

To evaluate our approach, the point clouds of five scenes obtained by a Faro Focus^3D^ TLS scanner on a campus were used. Five scenes contained 2,129,780 points, 2,021,938 points, 1,378,108 points, 2,077,624 points, and 1,524,230 points, respectively. We used one scene as the training set and the other four as testing sets. To train the classifier and evaluate the classification performance, we manually labelled the five scenes into the following six classes: ground, façade, pole-like object (note: we use pole for short in the following paper), tree, vegetation, and curb. The intensity values, RGB values, and ground truth labelling of the training set and four testing sets are illustrated in Figure 2. An unbalanced distribution of training examples per class may influence the training process [31]. Therefore, we used class re-balancing by randomly sampling the same number of training examples per class to acquire reduced training sets. In this study, we randomly selected a training set with 1000 training examples per class for the training set in one scene; a total of 6000 points were selected. All the points in the other four scenes were used as testing sets. The numbers of 3D points in six different classes of the training set and four testing sets are listed in Table 2.

### 3.2. Classification and Evaluation

The five data sets were first applied to the supervoxel generation. The resolution of supervoxels was set to 0.5 m for all data sets. After the supervoxel generation, the training set was over-segmented into 12,687 supervoxels, testing set 1 was over-segmented into 16,393 supervoxels, testing set 2 into 8470 supervoxels, testing set 3 into 15,240 supervoxels, and testing 4 into 12,591 supervoxels. 

**Figure 3:**
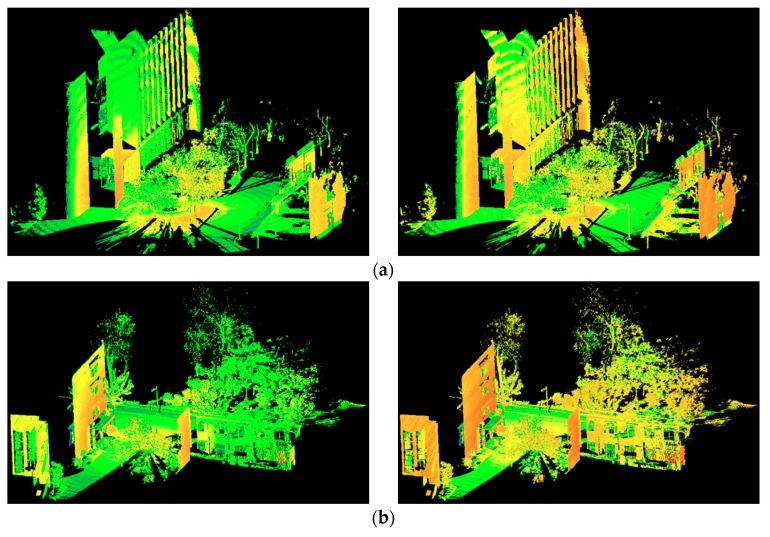

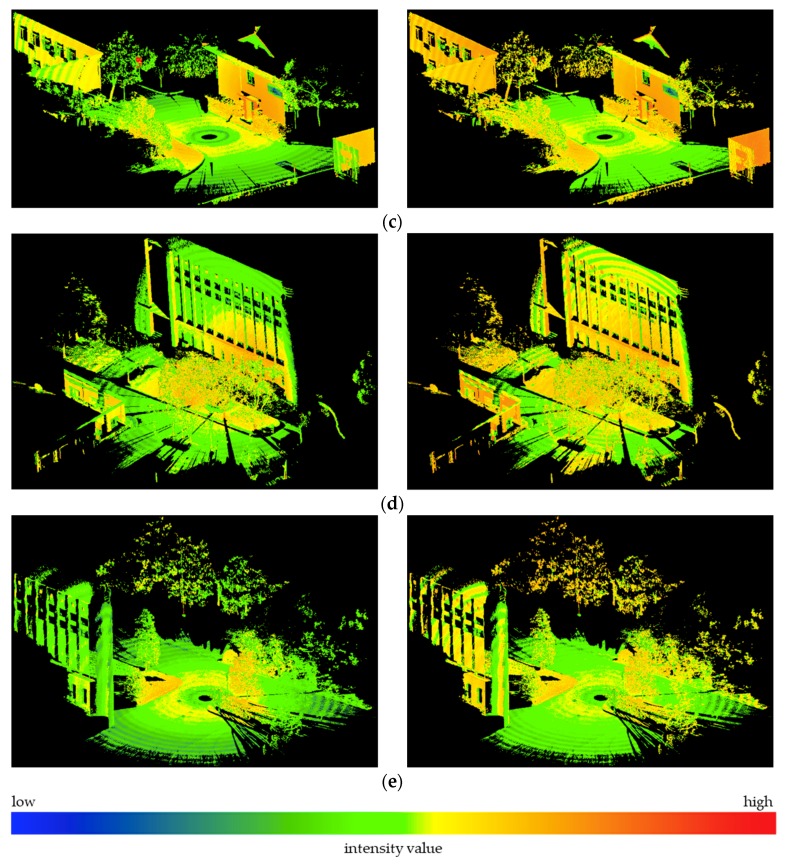
Original intensity (left) and corrected intensity (right) for: (**a**) training set, (**b**) testing case 1, (**c**) testing case 2, (**d**) testing case 3, and (**e**) testing case 4.

Afterwards, the intensity data of all training and testing point clouds were corrected for range effects before feature extraction. The specific form of the correction equation and the experiment conducted to derive the correction model can be found in our previous study [32]. The standard reference range was set to 5.0 m in this experiment. The visualization of the original and corrected intensity of all the data sets are shown in Figure 3, in which the intensity values are shown in pseudo color. The intensity values of the same object before correction vary a lot. As we can see from Figure 3, the intensity values are larger when the objects are closer to the scan station. After range effect correction, the intensity variation of the same object decreases a lot and the intensity variation among different objects increases.

**Figure 4:**
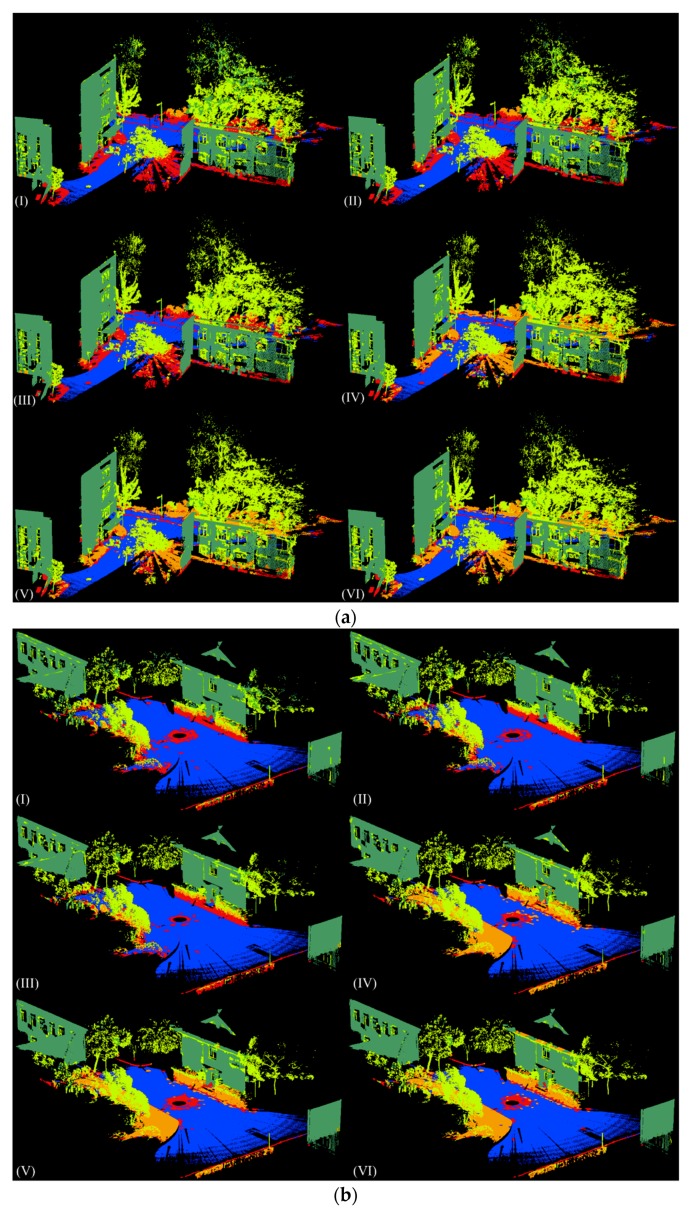

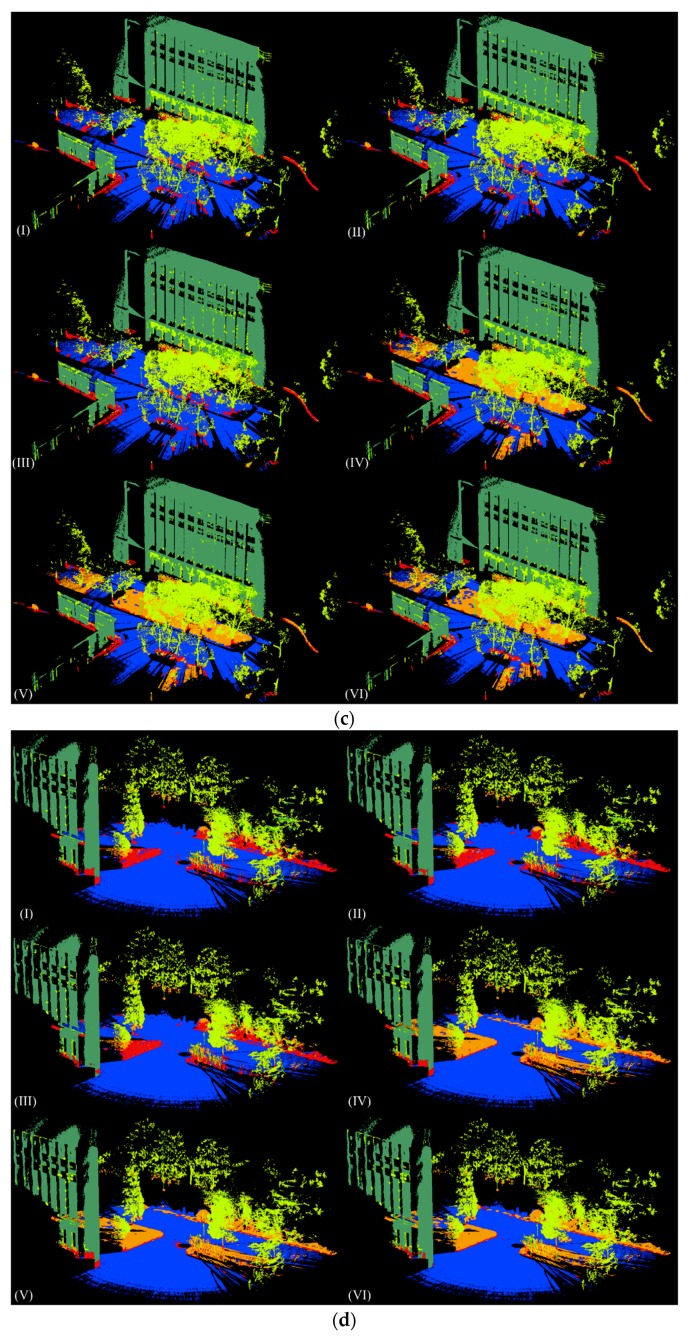
Visualization of the classification results of four testing sets: (**a**) testing set 1, (**b**) testing set 2, (**c**) testing set 3, and (**d**) testing set 4, obtained through six feature sets: (I) Geo, (II) Geo & OI, (III) Geo & CI, (IV) Geo & C, (V) Geo & C & OI, (VI) Geo & C & CI. Legend for labels: ground façade pole tree vegetation curb.

Afterwards, different features were extracted based on the supervoxel neighborhood for each data set. The Random Forest classifiers involved in our experiments were trained and tested using Matlab’s own implementation. A total of 100 trees were used for our application. The visualization of the classification results of the four testing sets are shown in Figure 4. For each testing set, the classification results of six different feature sets are illustrated. The overall accuracy, precision/recall, and F1 score values of all four testing sets for each different feature sets are listed in Table 3 and Table 4, respectively. In these tables and figures, Geo stands for geometric features, Geo & OI stands for geometric & original intensity features, Geo & CI stands for geometric and corrected intensity features, Geo & C stands for geometric and color features, Geo & C & OI stands for geometric, color, and original intensity features, and Geo & C & CI stands for geometric, color, and corrected intensity features. 

From the results, we can see that the overall accuracy of geometric features alone achieves the lowest value, whereas geometric features combined with color and corrected intensity features achieves the highest for all four test cases. The accuracy improves a little when the original intensity is combined with geometric features. This may be due to the fact that the original intensity is affected by several factors. After intensity correction, the accuracy has been further improved. However, compared with intensity features, color features seem to be more helpful for classification. The overall accuracy increases 8.3%, 8.9%, 15%, and 12.9% for testing sets 1, 2, 3, and 4, respectively, when color features are involved, compared with 4.0%, 1.6%, 1.3%, and 1.2% when corrected intensity features are involved. Moreover, as shown in Table 3, geometric features combined with both color and original intensity features do not guarantee an improvement in classification. For both testing set 3 and testing set 4, the overall accuracy decreases when original intensity features are involved, compared to when only color features are involved. After intensity correction, the overall accuracy for three features combined together increases a little and achieves the highest among six different feature sets. However, the increment is rather small, which may be due to the fact that only range effect correction has been conducted in this study. The intensity data is still affected by other factors like the incidence angle and environment etc. Further study will be focused on a more accurate intensity correction method.

**Figure 5:**
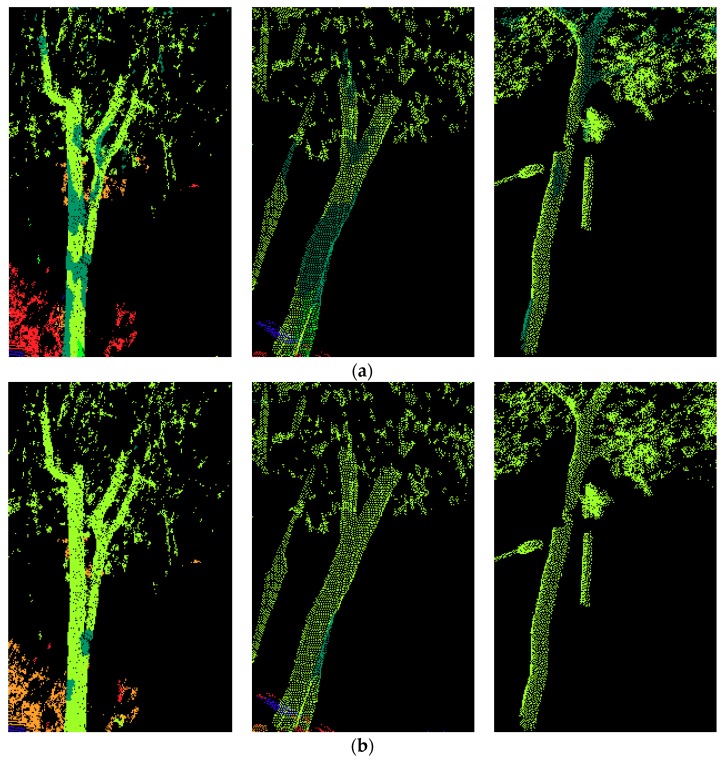
Examples of the misclassification between façades and trees with different feature sets: (**a**) Geo, (**b**) Geo & C.

The precision values differ a lot among different classes. A façade is detected with better precision compared to other classes, with over 90% for all three test cases. A façade has a relatively regular shape: only a few other points are misclassified as façades. Some tree points were wrongly classified as façade points, which may be because the tree trunks are similar in shape to some window frames on the facades. Furthermore, some trees were rather close to facades, which also lead to the misclassification between these two. The recall values of a tree were the lowest when only geometric features were used; after the combination with color features, the recall values achieved the highest values. Examples of the misclassification between façades and trees are shown in Figure 5. As we can see, when geometric and color features were used, fewer tree points were wrongly classified as façade points, compared with when only geometric features are used.

**Figure 6:**
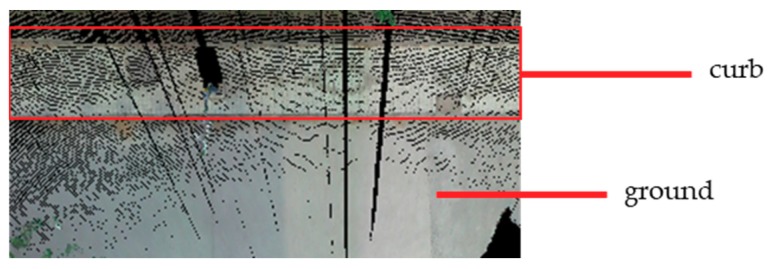
The similarity in color between ground and curb.

Grounds also achieved relatively high recall values for all four testing sets, because they have regular shapes and are the lowest in height among all six classes. Many curb points were wrongly classified as ground, because curbs are connected with the ground, and curbs are also in relatively low. In addition, the color and material of curbs and grounds in our test scenes are relatively similar, as shown in Figure 6. Therefore, additional color and intensity features could not help distinguish between these two. Some vegetation points were also wrongly classified as ground when only geometric features were used. This may be because some low vegetation is similar to the ground to some extent. When color features were added, fewer vegetation points were wrongly classified.

**Figure 7:**
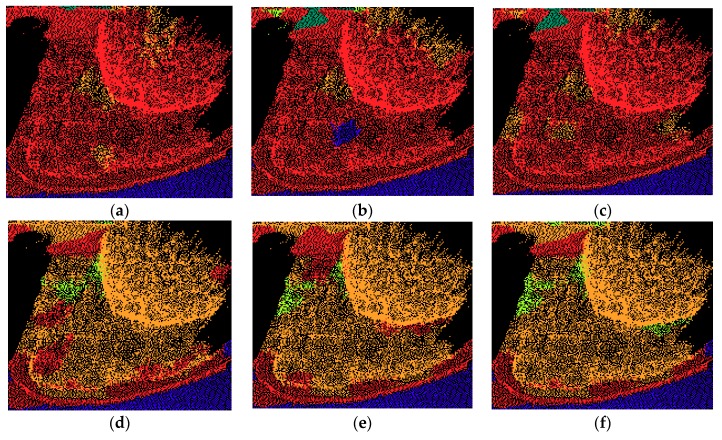
Examples of the misclassification of curbs with different feature sets: (**a**) Geo, (**b**) Geo & I, (**c**) Geo & CI, (**d**) Geo & C, (**e**) Geo & C & OI, (**f**) Geo & C & CI.

Curb had relatively low precision values in all four testing sets. This is mainly because the curbs were all connected with vegetation, and the geometric features of these two are similar to some extent. Therefore, many vegetation points were classified as curb points, which also lead to the low recall values of vegetation. When only geometric features were applied, lots of vegetation points were wrongly classified as curb points, as shown in Figure 7a. Note that when intensity features were used, the precision only slightly improved, as shown in Figure 7b,c. However, when color features were used, the precision value greatly improved, as shown in Figure 7d–f. In addition, precision was at its highest when geometric features were combined with color and corrected intensity features. Moreover, when additional color and intensity features were used, the precision of vegetation also improved.

**Figure 8:**
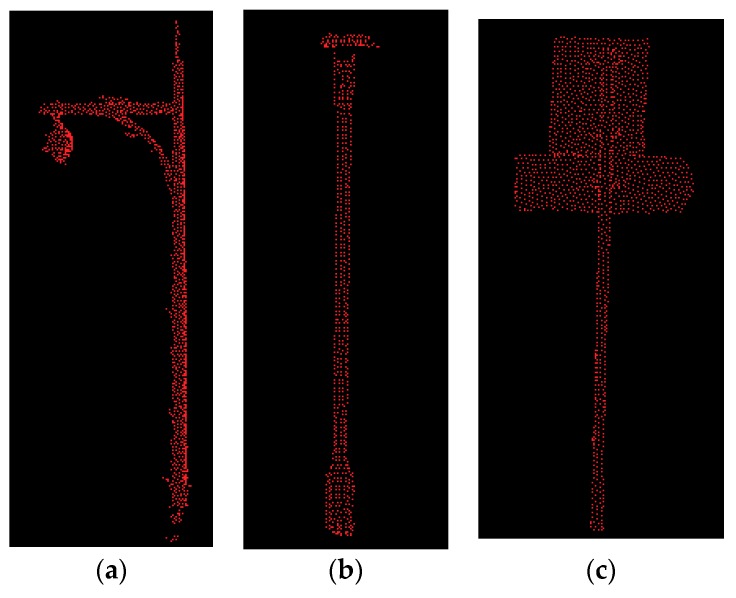
Different types of pole-like structures: (**a**) street lamp, (**b**) street lamp, (**c**) road sign.

The precision values of trees was relatively high among six classes. In testing set 1, 3, and 4, the average precision values were all above 80%. However, the precision values in testing set 2 was relatively low compared to other testing sets. This is mainly because the number of tree points in testing set 2 were smaller than other testing sets. From the results of all the testing sets, we found that additional intensity and color features could not guarantee the improvement of the precision of tree points. For some testing sets, additional intensity and color could even decrease the precision. This may be due to the fact that the tree leaves were similar to some vegetation in both color and intensity. The precision values of poles varied a lot among different testing cases as well as among different feature sets. This could be explained by the difference in poles: in four testing sets, poles included several different objects like street lamps and road signs. The shapes of different street lamps and road signs also varied a lot, as shown in Figure 8. Moreover, the shape of tree trunks was also similar to some poles, which would explain why some tree points were also wrongly classified as poles.

The recall values in this study varied a lot among six different classes. Among the six different classes, the ground was detected with best recall compared to the rest classes, with over 90% on average for all four testing sets. Because the ground is regular in shape, most ground points are classified correctly. Façades were detected with over 80% on average for all four testing sets, respectively. Because façades were also relatively regular in shape, they also had a great number of points. Additional color and intensity features were not successful in improving the recall values of these objects, as their regular shape can be classified correctly with only geometric features. Trees also achieved relatively high recall values. For all testing sets, when only geometric features were used, many tree points were wrongly classified as poles, due to their similar shape.

**Figure 9:**
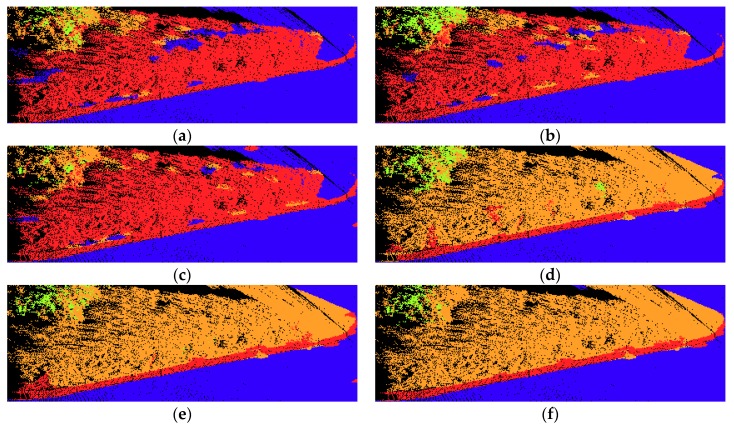
Examples of the misclassification of vegetation with different feature sets: (**a**) Geo, (**b**) Geo & I, (**c**) Geo & CI, (**d**) Geo & C, (**e**) Geo & C & OI, (**f**) Geo & C & CI.

Vegetation achieved relatively low recall values for all testing sets, with around 10% when only geometric features are used. This is mainly because vegetation is connected with curb, and they are partly similar in terms of geometric characteristics. Therefore, a great number of vegetation points were wrongly classified as curb points, and some were misclassified as ground points. As we can see from Figure 9a, when only geometric features are used, lots of vegetation points were classified as curb points. Additional intensity features did help a little in improving the classification, as seen from Figure 9b,c, and fewer vegetation points were misclassified as ground. Note that when color features were combined, most vegetation points were classified correctly. With additional intensity features, the classification results further improved, as shown in Figure 9e,f.

**Figure 10:**
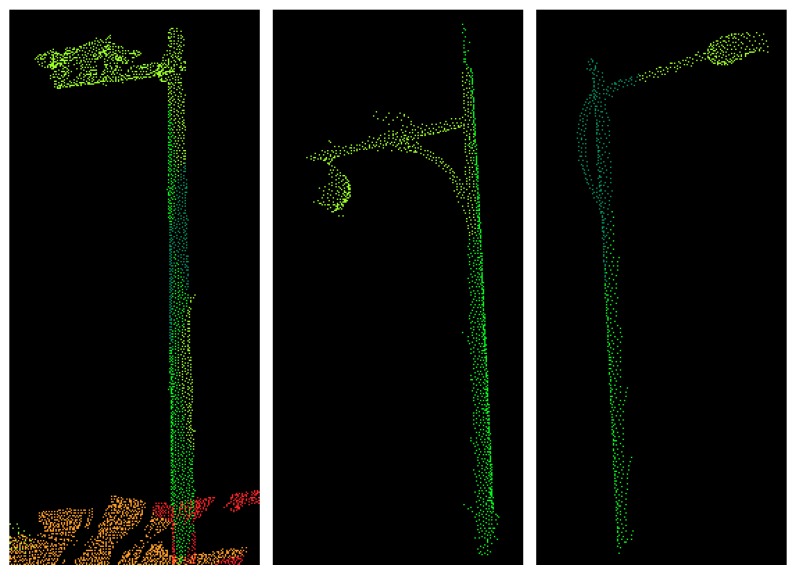
Examples of the misclassification of pole points.

Curbs also achieved relatively low recall values. This is mainly due to the connectivity between curbs and ground. Moreover, in all our testing sets, the curbs were similar to the ground in both color and materials. Therefore, additional color and intensity features may have decreased the classification accuracy instead of improving it. Poles achieved the lowest recall value in all four testing sets. Because in our testing sets, poles varied a lot (as shown in Figure 8), and the number of pole points was the smallest among six classes. Because different types of pole-like structures varied a lot in color and intensity, additional intensity and color features could not guarantee the improvement of classification results. Many pole points were classified as tree points because of the similarity in shape between these two. Moreover, some pole points were classified as façades, because some poles were also similar to some window frames to some extent. Examples of misclassification of pole points are illustrated in Figure 10.

## 4. Conclusions

In this paper, we carefully analyzed the performance of six different feature sets in TLS point cloud classification. A supervoxel neighborhood was used as a support region for feature extraction for the first time. Three types of features were then extracted and applied for classification using the Random Forest classifier. Experimental results demonstrate that both color and intensity features can help complement the geometric features to help improve the classification results in our test, especially for distinguishing between vegetation and curbs. Compared with intensity features, color features are more useful in TLS point cloud classification. Moreover, both the intensity data before and after correction were analyzed in this paper. The accuracy of corrected intensity features only slightly improved in our tests. This may be because in this study only range effect correction is performed. In further studies, a more accurate correction method will be developed for incidences of angle correction to derive a more accurate corrected intensity, thus further improving the classification results.

## Figures and Tables

**Table 1:** Three types of feature sets.

Geometric Features		Color and Intensity Features	
Linearity	$\left(\lambda_{1}-\lambda_{2}\right)/\lambda_{1}$	Mean R	$\left(\sum_{i=1}^{n}R_{i}\right)/n$
Planarity	$\left(\lambda_{2}-\lambda_{3}\right)/\lambda_{1}$	Mean G	$\left(\sum_{i=1}^{n}G_{i}\right)/n$
Sphericity	$\lambda_{3}/\lambda_{1}$	Mean B	$\left(\sum_{i=1}^{n}B_{i}\right)/n$
Omnivariance	${\left(\lambda_{1}\cdot \lambda_{2}\cdot \lambda_{3}\right)}^{1/3}$	R ratio	$\bar{R}/\sum_{i=1}^{n}(\bar{R}+\bar{G}+\bar{B})$
Anisotropy	$\left(\lambda_{1}-\lambda_{3}\right)/\lambda_{1}$	G ratio:	$\bar{G}/\sum_{i=1}^{n}(\bar{R}+\bar{G}+\bar{B})$
Eigenentropy	$-\sum_{i=1}^{3}\lambda_{i}\cdot \ln\left(\lambda_{i}\right)$	B ratio:	$\bar{B}/\sum_{i=1}^{n}(\bar{R}+\bar{G}+\bar{B})$
Sum of eigenvalues	$\lambda_{1}+\lambda_{2}+\lambda_{3}$	R variance	$\left(\sum_{i=1}^{n}R_{i}-\bar{R}\right)/n$
Change of curvature	$\lambda_{3}/\left(\lambda_{1}+\lambda_{2}+\lambda_{3}\right)$	G variance	$(\sum_{i=1}^{n}G_{i}-\bar{G})/n$
Mean Z	$\left(\sum_{i=1}^{n}Z_{i}\right)/n$	B variance	$\left(\sum_{i=1}^{n}B_{i}-\bar{B}\right)/n$
Z variance	$\left(\sum_{i=1}^{n}Z_{i}-\bar{Z}\right)/n$	Maximum R difference	$R_{max}-R_{min}$
Maximum Z difference	$Z_{max}-Z_{min}$	Maximum G difference	$G_{max}-G_{min}$
		Maximum B difference	$B_{max}-B_{min}$
		Mean intensity	$\left(\sum_{i=1}^{n}I_{i}\right)/n$
		Intensity variance	$\left(\sum_{i=1}^{n}I_{i}-\bar{I}\right)/n$
		Maximum intensity difference	$I_{max}-I_{min}$

**Table 2:** Number of 3D points in six different classes of training and testing sets.

	Training Set	Testing Set 1	Testing Set 2	Testing Set 3	Testing Set 4
Ground	387,419	285,096	450,805	234,590	579,463
Façade	1,061,658	732,092	478,830	1,006,474	322,725
Pole	5678	7230	6320	4183	4696
Tree	390,699	637,980	156,989	473,145	402,273
Vegetation	242,325	255,365	262,837	330,093	195,834
Curb	42,001	104,175	22,327	29,139	19,239
Total	2,129,780	2,021,938	1,378,108	2,077,624	1,524,230

**Table 3:** Overall accuracy (in %) of four test cases for different feature sets.

Feature Set	Testing Set 1	Testing Set 2	Testing Set 3	Testing Set 4
Geo	74.6	75.1	75.2	79.0
Geo & OI	76.0	75.1	76.2	80.2
Geo & CI	78.6	76.7	76.5	80.2
Geo && C	82.9	84.0	90.2	91.9
Geo && C && OI	83.8	84.0	89.8	91.8
Geo && C && CI	83.8	84.1	90.3	92.2

**Table 4:** Precision/Recall and F1 score values (in %) of four testing sets for different feature sets.

**Test Case 1**	**Ground**	**Façade**	**Pole**	**Tree**	**Vegetation**	**Curb**
Geo	76.2/98.4	90.2/83.6	16.6/25.6	88.1/86.8	63.9/11.3	11.0/30.8
	85.9	86.8	20.2	87.4	19.2	16.2
Geo & I	76.7/97.9	93.7/83.1	15.9/20.1	86.8/91.1	70.2/12.0	12.3/33.9
	86.0	88.1	17.8	88.9	20.5	18.1
Geo & CI	76.2/97.6	97.2/86.1	22.4/34.4	89.0/94.6	73.2/16.6	12.6/31.6
	85.6	91.3	27.1	91.7	27.1	18.0
Geo & C	80.8/95.2	99.0/77.0	61.0/24.7	81.1/97.4	76.0/74.5	27.9/26.1
	87.4	86.6	35.1	88.5	75.2	27.0
Geo & C & I	82.3/95.5	98.8/76.1	66.6/26.3	82.0/97.7	79.5/81.3	30.6/30.1
	88.4	86.0	37.7	89.1	80.4	30.3
Geo & C & CI	81.5/95.8	98.9/76.0	72.9/26.3	79.4/97.9	81.1/81.1	38.0/29.1
	88.1	86.0	38.6	87.7	81.1	32.9
**Test Case 2**	**Ground**	**Façade**	**Pole**	**Tree**	**Vegetation**	**Curb**
Geo	83.9/91.0	94.8/91.1	21.1/15.8	62.8/85.8	87.8/13.6	10.3/77.7
	87.4	92.9	18.1	72.5	23.5	18.1
Geo & I	80.9/93.3	97.0/88.8	42.0/14.6	58.2/92.0	89.6/10.1	12.5/78.5
	86.7	92.7	21.7	71.3	18.2	21.6
Geo & CI	82.3/96.3	98.0/88.4	33.8/21.2	60.4/93.4	84.6/13.1	13.1/76.9
	88.7	92.9	26.0	73.4	22.7	22.3
Geo & C	98.4/87.1	99.3/85.1	100.0/6.0	56.0/97.1	82.8/72.2	23.2/70.1
	92.4	91.6	11.3	71.1	77.1	34.9
Geo & C & I	99.1/86.5	99.2/84.3	100.0/6.0	56.4/97.2	87.7/74.4	19.1/71.8
	92.4	91.1	11.3	71.4	80.5	30.1
Geo & C & CI	98.9/88.5	99.3/81.8	100.0/6.0	56.1/96.4	81.8/76.3	24.2/71.5
	93.4	89.7	11.3	71.0	79.0	36.2
**Test Case 3**	**Ground**	**Façade**	**Pole**	**Tree**	**Vegetation**	**Curb**
Geo	50.6/99.8	99.1/84.8	4.5/15.9	78.0/94.1	38.5/3.8	12.5/58.6
	67.2	91.4	7.0	85.3	6.9	20.6
Geo & I	49.6/99.8	99.5/86.3	5.1/15.2	79.2/95.0	46.4/4.0	13.9/59.1
	66.3	92.4	7.6	86.4	7.4	22.5
Geo & CI	50.3/99.8	99.4/87.1	7.7/25.3	80.7/94.7	41.3/4.0	13.4/59.4
	66.9	92.9	11.9	87.1	7.2	21.9
Geo & C	87.4/99.8	99.7/89.4	11.8/29.3	83.6/96.8	95.1/79.6	24.5/60.9
	93.2	94.3	16.8	89.7	86.6	35.0
Geo & C & I	88.7/99.8	99.8/88.6	20.9/34.3	81.5/97.4	96.3/78.5	24.3/65.9
	93.9	93.9	26.0	88.7	86.5	35.5
Geo & C & CI	86.8/99.8	99.8/89.3	15.6.28.4	82.5/97.0	95.1/80.3	28.3/62.8
	92.9	94.2	20.2	89.1	87.1	39.0
**Test Case 4**	**Ground**	**Façade**	**Pole**	**Tree**	**Vegetation**	**Curb**
Geo	86.8/99.2	94.9/93.8	14.6/17.4	98.0/75.7	14.4/3.7	7.9/71.8
	92.6	94.4	15.9	85.4	5.9	14.2
Geo & I	86.8/99.0	96.0/94.2	15.2/17.7	97.7/80.4	20.1/3.6	8.1/73.2
	92.5	95.1	16.3	88.2	6.2	14.5
Geo & CI	86.9/99.5	97.5/95.5	17.5/23.8	97.6/77.6	17.4/4.9	8.8/74.2
	92.8	96.5	20.2	86.5	7.7	15.7
Geo & C	99.1/97.8	98.8/93.2	95.9/24.5	96.9/84.8	71.2/91.9	26.3/57.5
	98.4	95.9	39.1	90.4	80.2	36.1
Geo & C & I	99.0/97.0	98.7/92.7	97.2/28.2	96.5/85.3	72.9/92.7	24.4/60.6
	98.0	95.6	43.7	90.5	81.6	34.8
Geo & C & CI	97.9/98.2	98.6/94.7	97.7/33.9	96.2/84.9	71.5/90.2	39.1/60.1
	98.0	96.6	50.3	90.2	79.8	47.4

## References

[1] Weinmann M., Jutzi B., Hinz S., Mallet C. (2015). Semantic point cloud interpretation based on optimal neighborhoods, relevant features and efficient classifiers. ISPRS J. Photogramm. Remote Sens..

[2] Lim E.H., Suter D. (2009). 3D terrestrial LIDAR classifications with super-voxels and multi-scale Conditional Random Fields. Comput. Aided Des..

[3] Ramiya A.M., Nidamanuri R.R., Ramakrishnan K. (2016). A supervoxel-based spectro-spatial approach for 3d urban point cloud labelling. Int. J. Remote Sens..

[4] Wang H., Wang C., Luo H., Li P., Chen Y., Li J. (2015). 3-D point cloud object detection based on supervoxel neighborhood with Hough forest framework. IEEE J. Sel. Topics Appl. Earth Obs. Remote Sens..

[5] Plaza-Leiva V., Gomez-Ruiz J.A., Mandow A., García-Cerezo A. (2017). Voxel-Based Neighborhood for Spatial Shape Pattern Classification of Lidar Point Clouds with Supervised Learning. Sensors.

[6] Weinmann M., Hinz S., Weinmann M. (2017). A hybrid semantic point cloud classification–segmentation framework based on geometric features and semantic rules. PFG Photogramm. Remote Sens. Geoinf..

[7] Song J.H., Han S.H., Yu K.Y., Kim Y.I. (2002). Assessing the possibility of land-cover classification using LIDAR intensity data. Int. Arch. Photogramm. Remote Sens. Spat. Inf. Sci..

[8] Zhou W. (2013). An object-based approach for urban land cover classification: Integrating LiDAR height and intensity data. IEEE Geosci. Remote Sens..

[9] Zhang J.X., Lin X.G., Ning X.G. (2013). Svm-based classification of segmented airborne lidar point clouds in urban areas. Remote Sens..

[10] Guan H., Li J., Yu Y., Wang C., Chapman M., Yang B. (2014). Using mobile laser scanning data for automated extraction of road markings. ISPRS J. Photogramm. Remote Sens..

[11] Kumar P., McElhinney C.P., Lewis P., McCarthy T. (2014). Automated road markings extraction from mobile laser scanning data. Int. J. Appl. Earth Obs. Geoinf..

[12] Li L., Zhang D., Ying S., Li Y. (2016). Recognition and reconstruction of zebra crossings on roads from mobile laser scanning data. ISPRS Int. J. Geo-Inf..

[13] Hackel T., Wegner J.D., Schindler K. (2016). Fast semantic segmentation of 3D point clouds with strongly varying density. ISPRS Ann. Photogramm. Remote Sens. Spat. Inf. Sci..

[14] Tan K., Cheng X. (2015). Intensity data correction based on incidence angle and distance for terrestrial laser scanner. J. Appl. Remote Sens..

[15] Li Z., Zhang L., Tong X., Du B., Wang Y., Zhang L., Zhang Z., Liu H., Mei J., Xing X. (2016). A three-step approach for TLS point cloud classification. IEEE Trans. Geosci. Remote Sens..

[16] Aijazi A.K., Checchin P., Trassoudaine L. (2013). Segmentation based classification of 3D urban point clouds: A super-voxel based approach with evaluation. Remote Sens..

[17] Lin Y., Wang C., Zhai D., Li W., Li J. (2018). Toward better boundary preserved supervoxel segmentation for 3D point clouds. ISPRS J. Photogramm. Remote Sens..

[18] Papon J., Abramov A., Schoeler M., Worgotter F. Voxel cloud connectivity segmentation-supervoxels for point clouds. Proceedings of the IEEE Conference on Computer Vision and Pattern Recognition.

[19] Kang Z., Yang J. (2018). A probabilistic graphical model for the classification of mobile LiDAR point clouds. ISPRS J. Photogramm. Remote Sens..

[20] Hofle B., Pfeifer N. (2007). Correction of laser scanning intensity data: Data and model-driven approaches. ISPRS J. Photogramm. Remote Sens..

[21] Kashani A., Olsen M., Parrish C., Wilson N. (2015). A review of lidar radiometric processing: From AD HOC intensity correction to rigorous radiometric calibration. Sensors.

[22] Kaasalainen S., Jaakkola A., Kaasalainen M., Krooks A., Kukko A. (2011). Analysis of incidence angle and distance effects on terrestrial laser scanner intensity: Search for correction methods. Remote Sens..

[23] Fang W., Huang X., Zhang F., Li D. (2015). Intensity correction of terrestrial laser scanning data by estimating laser transmission function. IEEE Trans. Geosci. Remote Sens..

[24] Tan K., Cheng X. (2016). Correction of incidence angle and distance effects on tls intensity data based on reference targets. Remote Sens..

[25] Jelalian A.V. (1992). Laser Radar Systems.

[26] Demantké J., Vallet B., Paparoditis N. (2012). Streamed vertical rectangle detection in terrestrial laser scans for facade database production. ISPRS Ann. Photogramm. Remote Sens. Spat. Inf. Sci..

[27] Weinmann M., Jutzi B., Mallet C. (2014). Semantic 3d scene interpretation: A framework combining optimal neighborhood size selection with relevant features. ISPRS Ann. Photogramm. Remote Sens. Spat. Inf. Sci..

[28] Breiman L. (2001). Random forests. Mach. Learn..

[29] Chehata N., Guo L., Mallet C. (2009). Airborne LiDAR feature selection for urban classification using random forests. Int. Arch. Photogramm. Remote Sens..

[30] Hackel T., Wegner J.D., Schindler K. (2017). Joint classification and contour extraction of large 3D point clouds. ISPRS J. Photogramm. Remote Sens..

[31] Weinmann M., Weinmann M., Mallet C., Brédif M. (2017). A Classification-Segmentation Framework for the Detection of Individual Trees in Dense MMS Point Cloud Data Acquired in Urban Areas. Remote Sens..

[32] Li Q., Cheng X. (2018). Damage Detection for Historical Architectures Based on TLS Intensity Data. ISPRS Arch. Photogramm. Remote Sens. Spat. Inf. Sci..

